# HMGB1 Promotes the Release of Sonic Hedgehog From Astrocytes

**DOI:** 10.3389/fimmu.2021.584097

**Published:** 2021-04-01

**Authors:** Yifan Xiao, Yan Sun, Wei Liu, FanFan Zeng, Junyu Shi, Jun Li, Huoying Chen, Chang Tu, Yong Xu, Zheng Tan, Feili Gong, Xiji Shu, Fang Zheng

**Affiliations:** ^1^Department of Pathology and Pathophysiology, School of Medicine, Jianghan University, Wuhan, China; ^2^Department of Immunology, School of Basic Medicine, Tongji Medical College, Huazhong University of Science and Technology, Wuhan, China; ^3^School of Medicine, Institutes of Biomedical Sciences, Jianghan University, Wuhan, China; ^4^Wuhan Institute for Neuroscience and Neuroengineering, South-Central University for Nationalities, Wuhan, China; ^5^Department of Neurobiology, College of Life Sciences, South-Central University for Nationalities, Wuhan, China; ^6^Department of Laboratory Medicine, The Second Affiliated Hospital of Guilin Medical University, Guilin, China; ^7^Department of Orthopedics, Renmin Hospital of Wuhan University, Wuhan, China; ^8^Key Laboratory of Organ Transplantation, Ministry of Education, NHC Key Laboratory of Organ Transplantation, Key Laboratory of Organ Transplantation, Chinese Academy of Medical Sciences, Wuhan, China

**Keywords:** sonic hedgehog (shh), HMGB1, astrocytes, experimental autoimmune encephalomyelitis (EAE), receptor for advanced glycation end products (RAGE)

## Abstract

High mobility group box 1 protein (HMGB1) is known to be a trigger of inflammation in experimental autoimmune encephalomyelitis (EAE), an animal model of multiple sclerosis (MS). However, it may play a different role in some way. Here we investigated the effect of HMGB1 on promoting sonic hedgehog (shh) release from astrocytes as well as the possible signal pathway involved in it. Firstly, shh increased in astrocytes after administration of recombinant HMGB1 or decreased after HMGB1 was blocked when stimulated by homogenate of the onset stage of EAE. Moreover, the expression of HMGB1 receptors, toll-like receptor (TLR) 2 and receptor for advanced glycation end products (RAGE) increased after HMGB1 administration in primary astrocytes. However, the enhancing effect of HMGB1 on shh release from astrocytes was suppressed only after RAGE was knocked out or blocked. Mechanistically, HMGB1 functioned by activating RAGE-mediated JNK, p38, stat3 phosphorylation. Moreover, HMGB1 could induce shh release in EAE. Additionally, intracerebroventricular injection of recombinant shh protein on the onset stage of EAE alleviated the progress of disease and decreased demylination, compared to the mice with normal saline treatment. Overall, HMGB1 promoted the release of shh from astrocytes through signal pathway JNK, p38 and stat3 mediated by receptor RAGE, which may provide new insights of HMGB1 function in EAE.

**Grahpical Abstract:**
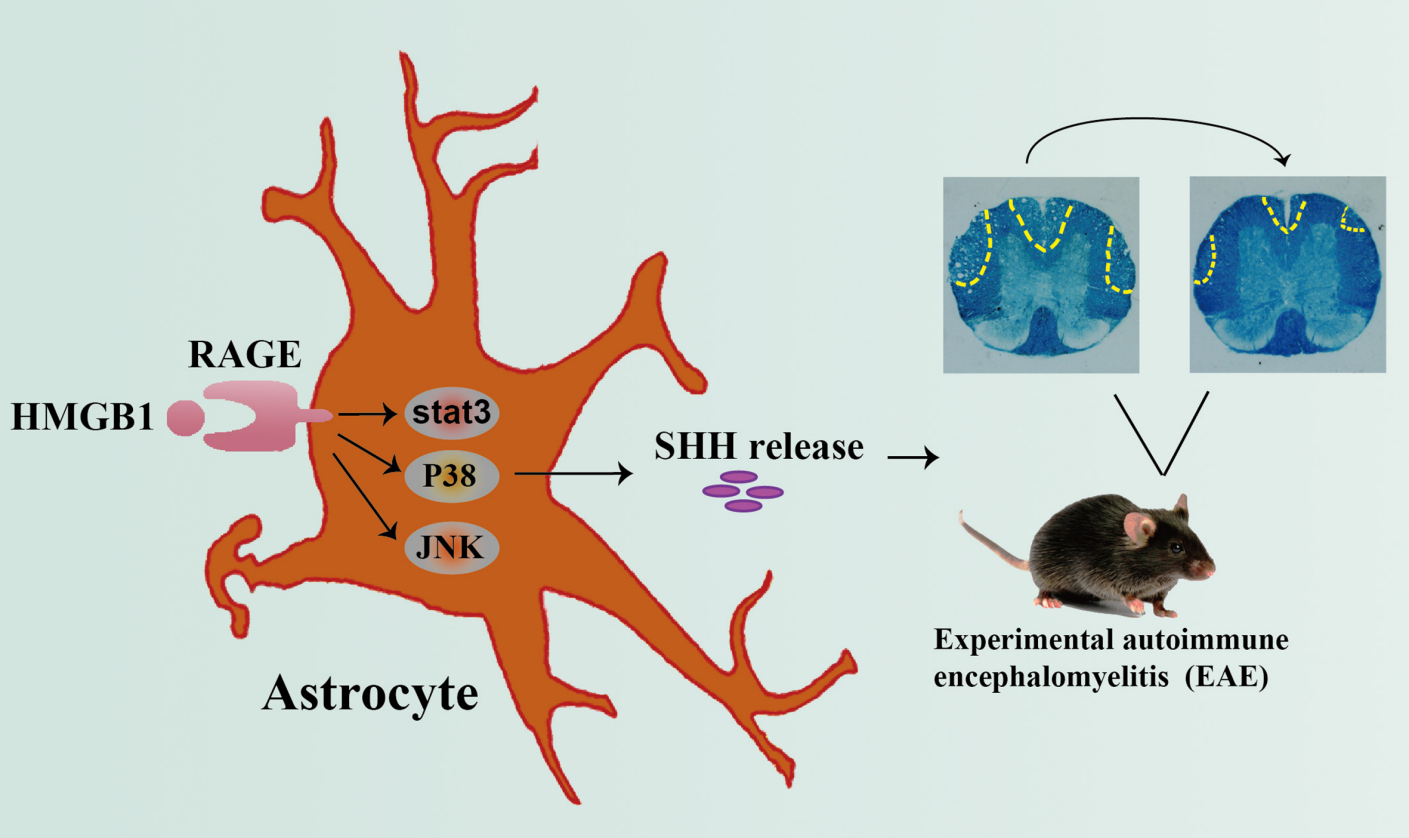
Graphical abstract depicting the effect of HMGB1 on promoting shh release from astrocytes through signal pathway JNK, p38 and stat3 mediated by receptor RAGE, which may provide new insights of HMGB1 function in EAE.

## Introduction

High-mobility group box 1 protein (HMGB1) is a typical damage-associated molecular pattern (DAMP) found in the nucleus of nearly all eukaryotic cells (1). In physiological conditions, HMGB1 is located in the nucleus, binding to DNA and participating in the transcription, replication, and repair of DNA. When cells die or get injured in disease, HMGB1 is released outside the cells, binding to different receptors, participating in innate or adaptive immune responses, and repairing damaged tissue (2–4). Moreover, the three main receptors implicated in HMGB1 are the receptor for advanced glycation end products (RAGE), toll-like receptor (TLR) 2 and TLR4 (5, 6). In recent years, HMGB1 has brought much interest for its pro-inflammatory role in diseases related to spinal cord injury, such as multiple sclerosis (MS) and related animal models-experimental autoimmune encephalomyelitis (EAE) (5). We previously confirmed that the level of HMGB1 in the brain of mice varied during different stages of EAE and became the highest on onset stage (7). The released HMGB1 in CNS can initiate neuro-inflammation and drive the progress of EAE (1).

Despite growing number of publications that describe pro-inflammatory effects of HMGB1, less is known about the role of HMGB1 in the repair of spinal cord injury (8). In fact, HMGB1 is also known as amphoterin, playing an important role in the early development of organism (9, 10). In zebrafish embryos, both forebrain neurons and brain size were significantly reduced after down-regulating HMGB1 expression (11). In cerebral ischemia, over-expression of HMGB1 in astrocytes promotes the repair of neurovascular units, while low expression of HMGB1 decreases the density of microvessels around the infarct and inhibits the repair of motor neurons (12).

The role of HMGB1 is flexible because its sulfhydryl in C23, C45 and C106 are easily oxidized by active oxygen in external environment. As reported, all-thiol-HMGB1 can form a complex with CXCL12. Then HMGB1-CXCL12 binds to CXCR4 and exerts its chemotaxis function. At the same time, the main biological effect of HMGB1 after binding to RAGE is promoting cells migration through the expression of adhesion molecules VCAM-1 and ICAM-1 or the secretion of CXCL12 (13, 14). Therefore, some studies indicate that all-thiol-HMGB1 mainly recognizes RAGE (15, 16). After the sulfydryl at C23 and C45 are oxidized to form a disulfide bond, disulfide (ds)-HMGB1 can specifically bind to myeloid differentiation factor 2 (MD2)-TLR4, promoting the secretion of chemokines and inflammatory factors (17, 18). Since HMGB1 may play different roles through different receptors, the exact role of HMGB1 in spinal cord injury remains to be further investigated. And until now, the underlying mechanism for the effects of HMGB1 remains unclear.

Astrocytes, the most abundant cell population in the central nervous system (CNS), are essential for normal neurological function. They respond to all forms of CNS damage and disease by undergoing cellular, molecular and functional changes. Astrocyte roles in CNS disorders exhibit diversity and a better understanding of this diversity has the potential to impact on the understanding and treatment of CNS injury and disease (19). In MS/EAE, astrocytes not only recruit lymphocytes and contribute to tissue damage but also confine inflammation and promote lesion repair (20). Understanding the emerging roles of astrocytes in MS/EAE will open up a new therapeutic opportunity.

Sonic hedgehog (Shh), a highly conserved secreted glycoprotein, is a member of the Hedgehog protein family in vertebrates (21, 22). It can activate the transcription of downstream target genes by downstream signaling pathway (21, 22). In MS/EAE, shh was upregulated in astrocytes and involved in promoting blood brain barrier (BBB) integrity or supporting neural stem cell (NSC) differentiation toward neurons and oligodendrocytes, facilitating remyelination and controlling axon growth (23–26). These results affirmed the important role of shh in improving functional recovery in MS/EAE. But the regulation mechanism of shh release is deficient. Among the limited researches, *Hmgb1* gene was reported to regulate the embryonic development partly by Shh signaling. However, the study was performed in the level of *Hmgb1* gene and non-pathological status. Together with the role of HMGB1 and Shh in neural injury (8, 25, 27), the limited understandings drive us to further characterize the relation of HMGB1 and shh. To validate the hypothesis, we design the experiment to investigated the relationship of HMGB1 and shh as well as the possible signal pathways involved in it.

## Material and Methods

### Ethics Statement

All animal experiments in this study were performed in strict accordance with the Institutional Animal Care and Use Committee, Tongji Medical College, Huazhong University of Science and Technology. All efforts were made to minimize animal suffering.

### Mice

Wild type (WT) C57BL/6 mice were purchased from SLAC Laboratory Animal Co. Ltd. (Shanghai, China) and maintained in a SPF facility for further used. TLR2^-/-^ mice were purchased from Nanjing Biomedical Research Institute of Nanjing University (Nanjing, China). TLR4^-/-^ mice were given as a gift from Hui Wang professor in Tongji Medical College, Huazhong University of Science and Technology, Wuhan, Hubei, PR China. RAGE^-/-^ mice were made by the loxp/cre recombinase system. RAGE^Loxp/Loxp^ and cre mice were given as a gift from Chongyi Wang professor in the Organ Transplantation Institute of Tongji Hospital, Tongji Medical College, Huazhong University of Science and Technology, Wuhan, Hubei, PR China.

### EAE Induction and Clinical Evaluation

Female C57BL/6J mice (8-9-week-old) were used for active induction of EAE, as described previously (7, 28, 29). Briefly, the mice were subcutaneously (s.c.) immunized with 200µg of MOG35-55 (CL Bio-Scientific Co. LTD., Xian, China), emulsified in Freund’s complete adjuvant containing 5 mg/mL of Mycobacterium tuberculosis (strain H37Ra; Difco Laboratories, Detroit, MI, USA). In addition, 200 ng pertussis toxin (Sigma, St. Louis, MO, USA) was intraperitoneally (i.p.) injected at day 0 and 2 post immunization. The mice were scored daily, according to the clinical symptoms. The criteria were as follows: 0, asymptomatic; 0.5, loss of the distal half of tail tone; 1, loss of entire tail tone; 2, hind limb weakness; 2.5, hind limb paraplegia; 3, both hind limb paraplegia;3.5, forelimb weakness and hind limb paraplegia; 4, forelimb and hind limb paraplegia; 5, moribund or death.

### GL Administration

The treatment of glycyrrhizin (Minophagen Pharmaceutical Co., Tokyo, Japan) was described previously (30). Briefly, a single intra-peritoneal (i.p.) dose of 25 mg/kg glycyrrhizin (GL) was administrated every other day from days 12 to 22 after induction of EAE.

### Interstitial Fluid Preparation

Brains from EAE were weighed and homogenized in sterile PBS (100 mg tissue per 1 mL of 1×PBS) containing a protease inhibitor 4-(2-Aminoethyl) benzenesulfonyl fluoride hydrochloride (AEBSF, 0.1mM, Sigma, St. Louis, MO, USA) on ice, then centrifuged at 12000 rpm for 20 min at 4°C. The supernatant was removed in a new tube and the protein concentration was detected using the BCA Protein Assay Kit (Thermo Fisher).

### Intracerebroventricular Injection

The procedure was performed as described previously (29). Briefly, after anesthetized intraperitoneally, a 26-gauge guide cannula (RWD life science, Shenzhen, China) was oriented into the left lateral ventricle using the following coordinates from Bregma: 0.5 mm posterior, 1.0 mm lateral, and 2.0 mm ventral. The guide cannula was secured by dental cement, anchored by stainless steel screws fixed to the skull, and sealed with a stainless steel wire to prevent occlusion. EAE induction was conducted 7 days later. During intracerebroventricular injection, a 30-gauge injection cannula connected to a 10-μL Hamilton syringe by a PE-20 catheter was filled with drug solution and inserted into the guide cannula extending 0.5 mm beyond the guide cannula tip. Recombinant mouse sonic hedgehog (Shh) protein (R&D system, Minneapolis, MN, USA) in 10 μL saline or 10 μL saline was delivered over a 2-min period every day from day 11 to day 19 post immunization.

### The Culture of Primary Astrocytes

The primary astrocytes were obtained from cerebral cortices of C57BL/6 mouse pups (P1-P2) as described previously (29, 31). Briefly, cerebral cortices were isolated, minced, and digested in 0.25% trypsin–0.01% EDTA for 45 minutes. Then 10% FBS was used to terminate the digestion and the samples were passed through a 70μm filter. The mixed cortical cells were plated in 25 cm^2^ (T25) culture flasks with DMEM/F12 medium (Gibco, Waltham, MA, USA) supplemented with 10% FBS (NATOCOR, Córdoba, Argentina) and cultured at 37°C in 5% CO_2_. Medium was changed one day after plating the mixed cortical cells and all 3 days thereafter. After 7 to 8 days, when astrocytes reached confluence, the T25 flasks were shaked at 250 rpm for 24h on an orbital shaker to remove microglia and oligodendrocyte precursor cells (OPC). Floating cells were removed and the remaining confluent astrocyte layer were digested by 0.25% trypsin–0.01% EDTA and plated in T75 culture flasks. 12-14 days after the first split, astrocytes were plated in an appropriate cell concentration 24 hour before performing the experiment. The purity of astrocytes was identified by immunofluorescence and flow cytometry.

### Flow Cytometry

After digested by 0.25% trypsin–0.01% EDTA and washed by 1×PBS-0.5%BSA, cells were incubated with anti-CD16/32 (BD Bioscience) for 15 minutes at room temperature to block the Fcγ receptors.

#### Staining of Cell Surface Antigens

All cells were resuspended in 1×PBS-0.5%BSA containing adequate antibody and incubated for 45 minutes at room temperature. The cells were then centrifuged for 5 min at 1000 rpm after washed by cold 1×PBS-0.5%BSA.

#### Intracellular Staining

The cells were permeabilized with Perm/Fix solution (BD Bioscience) and incubated with mouse anti-GFAP for 45min at room temperature. This was followed by a 30-min secondary incubation in FITC-anti mouse IgG (all diluted in 1×permwash). Cells were then washed by cold 1×PBS-0.5%BSA prior to FACS analysis by BD LSR II (BD Biosciences).

### Astrocytes Treatment

Astrocytes were plated at 1.7×10^5^ cells/well in 48-wells culture plates and cultured for 18h-24h before experiment. Then they were stimulated with HMGB1 (1-2μg/mL, Sigma, St. Louis, MO, USA) or interstitial fluid (100μg/mL) with/without the combination of antibody (HMGB1 Ab or IgG: 5 μg/mL) for 24 h. The culture supernatants were collected for ELISA while the remaining cells were performed for the following RT-PCR or Western blot assay.

Blocking agents for TLR4 (100nM TAK-242, Milipore, Temecula, CA, USA) or RAGE (148nM FPS-ZM1, Milipore, Temecula, CA, USA) in 10% FBS-DMEM/F12 medium were incubated with astrocytes for 2h at 37°C, 5%CO2 incubator. Inhibitor agents for p38 (3μM SB203580, MedChemExpress, Monmouth Junction, NJ,USA), ERK (2μM SCH772984, MedChemExpress, Monmouth Junction, NJ,USA), JNK (5μM SP600125, MedChemExpress, Monmouth Junction, NJ,USA) and stat3 (10μM SH-4-54, MedChemExpress, Monmouth Junction, NJ,USA) in 10% FBS-DMEM/F12 medium were incubated with astrocytes for appointed time at 37°C, 5%CO_2_ incubator. Subsequently appropriate HMGB1 were used to reach to the final concentration 1-2μg/mL. The remaining steps are the same as before.

### ELISA

The level of shh in the cell culture medium of astrocytes was measured by a mouse ELISA kit (R&D system, Minneapolis, MN, USA) according to the manufacturer’s instructions.

### Western Blot

The cells were lysed in RIPA buffer containing protease and phosphatase inhibitors on ice. The lysed cells were collected by cell scraper and then centrifuged for 30 min at 12000 rpm, 4°C. After that, the concentration of protein in supernatant was quantified using BCA Protein Assay Kit. Finally protein samples were mixed with 5 × SDS loading buffer, boiled for 5 min and stored in -80°C for further use.

Spinal cords from EAE were washed for three times using 1×PBS on ice to remove superficial blood, then single cell suspension were obtained after mechanical shear and grind in 1×PBS on ice followed by centrifuging (4000 rpm, 5 min, 4°C). The cell deposits were gathered and washed by 1×PBS on ice followed by centrifuging (4000 rpm, 2 min, 4°C). After that, cytoplasmic protein and nuclear protein were separated by cytoplasmic and nuclear protein extraction kit (Beyotime Biotechnology, ShangHai, China) according to the manufacturer’s instructions.

The protein was separated by 10%-12% SDS–PAGE and blotted onto polyvinylidene fluoride (PVDF) membranes (Hybond Inc., Escondido, CA, USA) as described (7). Blots were visualized by an ECL system (Pierce Bio-Technology, Rockford, IL) after incubating with horseradish peroxidase (HRP) conjugated secondary antibody (Thermo, Massachusetts, USA), and quantified by densitometry using the Biorad GelDoc XR Image analysis system (Bio Rad, Hercules, California, USA).

### Antibodies

Primary antibodies include rat anti-shh antibody (Abcam, UK), and mouse anti-β-actin antibody (EASYBIO, Beijing, China), mouse anti-GAPDH antibody (EASYBIO, Beijing, China), goat anti-RAGE polyclonal antibody (R&D system, USA), rabbit anti-AGER polyclonal antibody (Abcam, UK), rat anti-shh polyclonal antibody (Abcam, UK), mouse anti-GAPDH monoclonal antibody (EASYBIO, Beijing, China), mouse anti-GFAP monoclonal antibody (Millipore, USA), mouse anti-HMGB1 monoclonal neutralizing antibody (Institute of Biophysics, Chinese Academy of Sciences, Beijing, China), mouse IgG (Santa Cruz, USA), rabbit anti phosphorylation-ERK antibody (CST, USA), rabbit anti phosphorylation-p38 antibody (CST, USA), rabbit anti phosphorylation-JNK antibody (CST, USA), rabbit anti phosphorylation-stat3 antibody (Abcam, UK), rabbit anti total-ERK antibody (CST, USA), rabbit anti total-p38 antibody (CST, USA), rabbit anti total-JNK antibody (CST, USA), rabbit anti total-stat3 antibody (Abcam, UK). The antibodies for flow cytometry include PE-anti-mouse-TLR2 (eBioscience, San Diego, CA; clone: 6C2), PE-cy7-anti-mouse-TLR4 (Biolegend, San Diego, USA; clone: MTS510).

### Statistical Analysis

Experimental data are expressed as the mean ± standard deviation (SD). The data for more than two groups was analyzed with one-way analysis of variance (ANOVA) followed by Tukey’s multiple comparison test. Other data were analyzed using two-tailed unpaired Student’s t-test. A *P* value of <0.05 was considered to be statistically significant (*P< 0.05, **P< 0.01, ***P< 0.001).

## Results

### HMGB1 Promotes the Expression and Release of Shh in Primary Astrocytes

**Figure 1:**
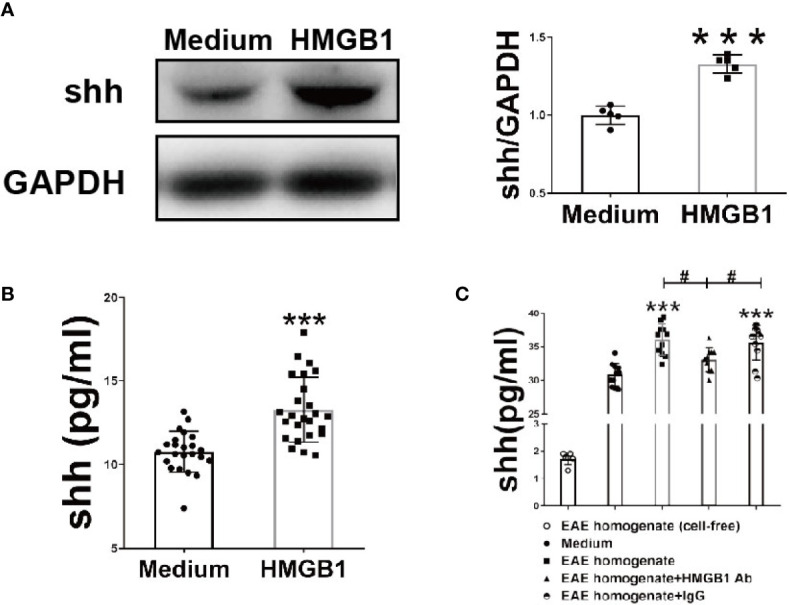
The effect of HMGB1 on expression and release of shh in astrocytes. **(A)** The protein from astrocytes was obtained and detected by western blot (1μg/ml recombinant HMGB1 was used). **(B)** The level of shh in supernatant from astrocytes after recombinant HMGB1 (1μg/ml) or **(C)** brain homogenate of EAE onset stage (100μg/ml) with/without HMGB1 Ab/IgG (5μg/ml) stimulation for 24h were detected by ELISA. EAE homogenate (cell-free) group here indicates interstitial fluid (100μg/ml) from the onset stage of EAE mice without cultured astrocytes. All the data are shown as mean ± SD (***P < 0.001 compared with medium; ^#^*P* < 0.05 compared with each other).

To investigate the effects of HMGB1 on shh expression and release in astrocytes, western blot analysis and ELISA were performed. The expression and release of Shh by astrocytes significantly increased, after HMGB1 (1μg/ml) stimulation for 24h (**Figures 1A, B**). There was no significant change in cell viability after HMGB1 stimulation, and cell survival controls have been supplied in **Supplemental Figure 1**. To simulate the microenvironment in EAE, we obtained the interstitial fluid of brain from the onset stage of EAE, during which the level of HMGB1 reached to the highest as described in our previous study (7). We found that shh levels increased in astrocytes when simulated by the interstitial fluid (100μg/ml) compared to medium group. And the effect was reversed after HMGB1 antibody (5μg/ml) treatment. Moreover, when treated with control IgG (5μg/ml) instead of HMGB1 antibody, the shh levels remained unchanged (**Figure 1C**). It is worth mentioning that shh in EAE homogenate (cell-free) group was less than 2pg/ml, which was much lower than that of the medium group. The shh in EAE homogenate would not affect the result. The data above indicated that HMGB1 could promote shh expression and release in astrocytes.

### The Effect of HMGB1 on Shh Release From Astrocytes Is Mainly Through Receptor RAGE

**Figure 2:**
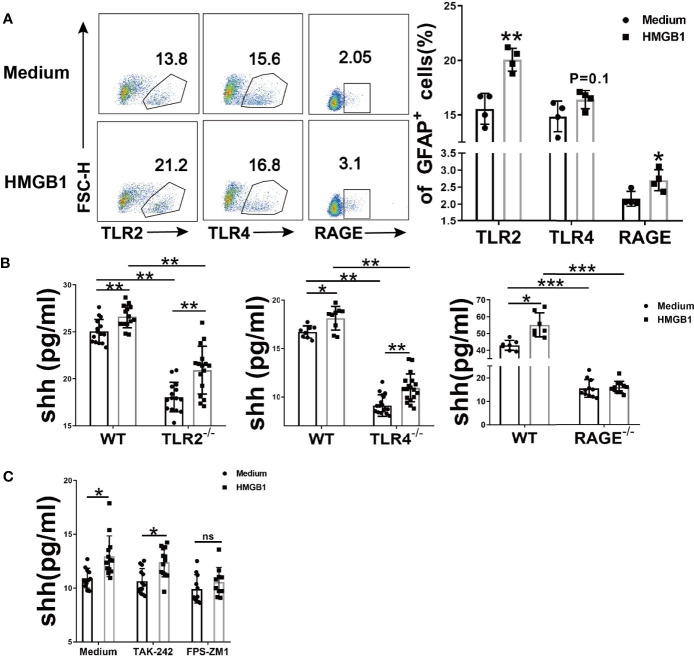
The effect of HMGB1 receptors on the release of shh in astrocytes. **(A)** Three surface receptors for HMGB1 were analyzed by flow cytometry after HMGB1 stimulation (2μg/ml) in astrocytes. **(B)** The release of shh from astrocytes was detected after TLR2, TLR4 and RAGE were knocked out (2μg/ml and 1μg/ml recombinant HMGB1 was used in TLR2, TLR4 and RAGE knockout astrocytes respectively). **(C)** The effect of TLR4 blocker (TAK-242: 100nM) and RAGE blocker (FPS-ZM1: 148nM) on the release of shh in astrocytes (1μg/ml recombinant HMGB1 was used). Data are shown as mean ± SD (*P < 0.05, **P < 0.01, ***P < 0.001). ns, no significance.

To investigate which receptor is changed in astrocytes after HMGB1 (2μg/ml) stimulation, flow cytometry was performed. The surface TLR2 and RAGE increased significantly while TLR4 remained unchanged (**Figure 2A**). Considering that TLR4 may internalized into endosomes after stimulation (32, 33), we checked the change of total TLR4 protein in astrocytes. The result demonstrated that total TLR4 protein significantly increased in astrocytes after HMGB1 (2μg/ml) stimulation (**Supplemental Figures 2A, B**). Although HMGB1 receptors increased, it could not illuminate which receptor is crucial for the effect of HMGB1 on shh release from astrocytes.

To further explore the possible mechanism for shh release, we next knocked out TLR2, TLR4 and RAGE respectively. Compared to wild type astrocytes, the levels of shh in TLR2^-/-^, TLR4^-/-^ and RAGE^-/-^ astrocytes decreased (**Figure 2B**). We found that spontaneous shh release in astrocytes existed in medium group, the phenomenon reminded us spontaneous shh release was dependent on TLR2, TLR4 and RAGE. But as for HMGB1, the effect on shh release was weakened only after receptor RAGE was knocked out. To further prove this conclusion, we next introduced the blocking agents FPS-ZM1 and TAK-242, which could block RAGE and TLR4 respectively. As shown in **Figure 2C**, the levels of shh increased under the stimulation of HMGB1 (1μg/ml) comparing to that in medium group when TLR4 was blocked. But the levels of shh had no statistically significant increase after HMGB1 stimulation comparing to that in medium group when RAGE was blocked. It implied that HMGB1 promoted shh release in astrocytes mainly through receptor RAGE.

### p38, JNK, and stat3 Are Involved in the Effect of HMGB1 on Promoting Shh Release From Astrocytes Through Receptor RAGE

**Figure 3:**
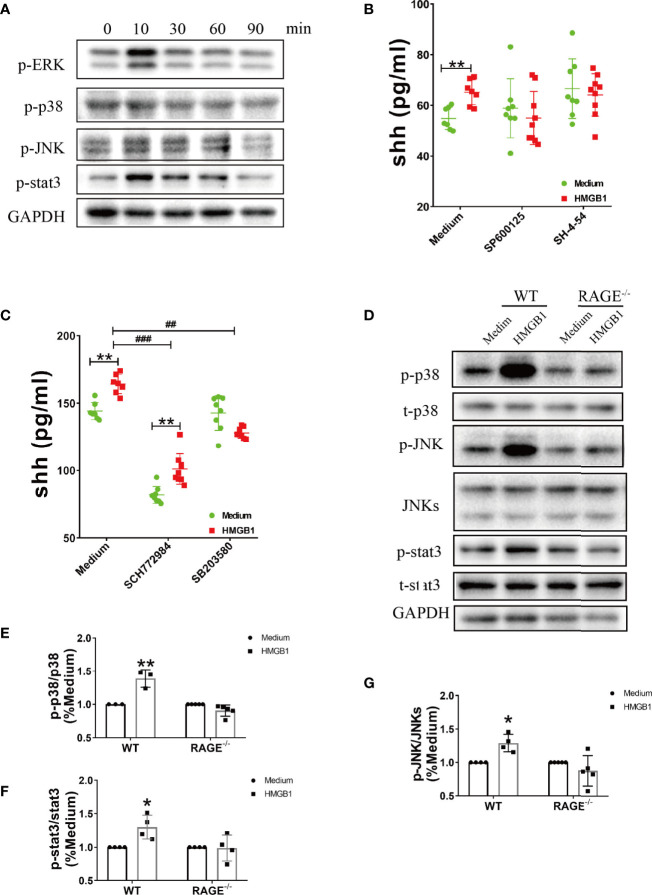
The signal pathways involved in the effect of HMGB1 on promoting shh release from astrocytes through receptor RAGE. **(A)** The change of phosphorylation-ERK, p38, JNK and stat3 after HMGB1 (1μg/ml) stimulation. **(B, C)** The effect of JNK blocker (5μM SP 600125), stat3 blocker (10μM SH-4-54), ERK blocker (2μM SCH 772984) and p38 blocker (3μM SB 203580) on shh release from astrocytes after HMGB1 (1μg/ml) stimulation for 10 min. **(D)** The change of phosphorylation-p38, JNK and stat3 after HMGB1 (1μg/ml) stimulation for 10 min in RAGE^-/-^ astrocytes comparing to WT astrocytes. **(E–G)** Data analysis for panel **(D)**. Data are shown as mean ± SD (*P < 0.05, **P < 0.01 compared with medium; ^##^*P* < 0.01, ^###^*P* < 0.001 compared with each other).

The downstream signal pathway for RAGE included MAP kinases (p38, ERK, JNK) and stat3. In our results, the phosphorylation of p38, ERK, JNK and stat3 significantly increased after HMGB1 (1μg/ml) stimulation for 10 minutes (**Figure 3A**, **Supplemental Figure 3**). We next explored the effect of inhibitors for stat3 and MAPK on shh release from astrocytes. As shown in **Supplemental Figure 4**, 5μM SP600125 (JNK blocker), 10μM SH-4-54 (stat3 blocker), 2μM SCH772984 (ERK blocker), 3μM SB203580 (p38 blocker) were chosen according to our preliminary experiment. When signal pathway JNK and stat3 were blocked, the level of shh had no significant change after HMGB1 (1μg/ml) stimulation for 10min. (**Figure 3B**). At the same time, when signal pathway ERK was blocked, shh release from astrocytes decreased, but the level of shh could still increase after HMGB1 (1μg/ml) stimulation for 10 min. When signal pathway p38 was blocked, shh release from astrocytes decreased and the level of shh did not increase after HMGB1 stimulation (1μg/ml) for 10 min (**Figure 3C**). The results indicated that the signal pathway involved in shh release after HMGB1 stimulation were closely related to stat3, JNK and p38. However, whether the above signal pathways depend on RAGE remained unknown. To furtherly illuminate it, we next detected the change of p-p38, p-JNK and p-stat3 in RAGE^-/-^ astrocytes. The result displayed that phosphorylation of p38, JNK and stat3 in RAGE^-/-^ astrocytes were weakened after HMGB1 (1μg/ml) stimulation for 10 min (**Figures 3D–G**). The above data revealed that the effect of HMGB1 on promoting shh release from astrocytes was through signal pathway stat3, JNK and p38 mediated by receptor RAGE.

### HMGB1 Promote Shh Expression in EAE

**Figure 4:**
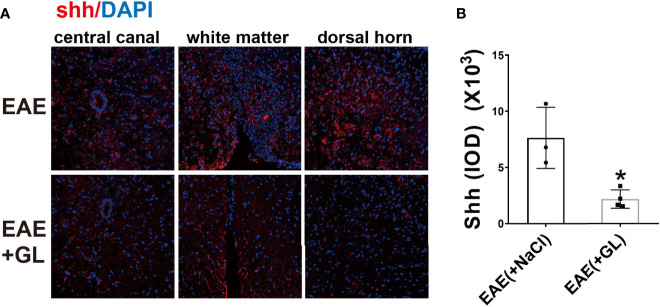
The effect of HMGB1 inhibitor (glycyrrhizin, GL) on the expression of shh in EAE. GL (25mg/kg) was injected intraperitoneally (i.p.) on the onset stage of EAE and the spinal cord tissues were collected on the peak stage. **(A)** Immunofluorescence was used to detect the expression of shh in CNS. Images are representative of 3 or 4 mice in each group and **(B)** data are shown as mean ± SD. (*P < 0.05).

However, whether HMGB1 could induce shh expression in EAE is unknown. Next we use glycyrrhizin (GL), a HMGB1 inhibitor, to further detect it *in vivo*. Based on our previous study (34), dose of 25 mg/kg glycyrrhizin (GL) was used in this study. As shown in **Figure 4**, GL (25mg/kg) treatment by intraperitoneal (i.p.) on the onset stage of EAE restrained shh expression compared to normal saline group (**Figures 4A, B**). The data indicated that HMGB1 could promote shh expression in EAE.

### Shh Treatment Alleviated the Progress of EAE

**Figure 5:**
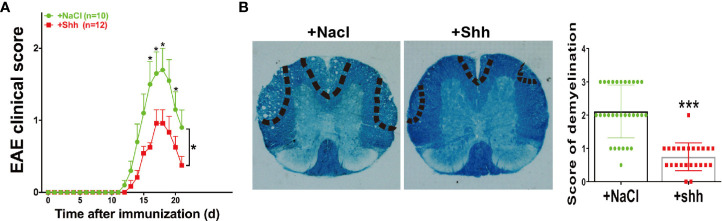
The effect of shh on the progress of EAE. **(A)** Shh protein treatment was applied on the onset stage of EAE and the clinical score was observed across the progress of EAE. Data are shown as mean ± SEM. **(B)** LFB staining was used to study the demyelination of the spinal cord sections. The sections were obtained on the remission stage of EAE (images are representative of 4-5 mice in each group). The bar denotes 200 μm. Data (n = 3-5 mice in each group, and 5–6 sections from each mouse were used for LFB staining and scoring) are shown as mean ± SD. (*P < 0.05, ***P < 0.001).

In MS/EAE, shh can act on endothelial cells to repair blood brain barrier (BBB). In our study, the progress of EAE was alleviated when recombinant shh protein by intracerebroventricular injection was performed from onset to peak stage of EAE (**Figure 5A**). Besides, demyelination during chronic stage was ameliorated in shh group, compared to saline group (**Figure 5B**). The data indicated that shh played a protective role in EAE.

## Discussion

Multiple sclerosis (MS) and related animal models-experimental autoimmune encephalomyelitis (EAE) is an autoimmune disease of the central nervous system (CNS), during which damaged neurons can release large amounts of HMGB1 (7). In 1999, Wang et al. found that HMGB1 played an important role as a late mediator of endotoxin lethality (35). Since then, numerous researches focused on the pro-inflammatory effects of HMGB1 in various diseases, including MS/EAE. However, increasing studies indicated that DAMPs are not only dangerous signals after tissue damage, but also proteins that can repair tissue (9, 13, 14). It has been demonstrated that HMGB1 can promote the regeneration of new tissues by recruiting stem cells after its pro-inflammatory activity in some diseases (36–39). Therefore, the exact role of HMGB1 remains to be further explored.

In MS/EAE, abundance of immune cells infiltrate into CNS, accompanied by demyelination, axonal damage, and local inflammation (40). As we know, the peripheral immune cells can infiltrate into CNS only when blood brain barrier (BBB) was damaged (41). The BBB is composed of capillary endothelial cells, basement membrane, and glial boundary membrane (the terminal protuberance of astrocytes adhere to the capillary wall to form a glial boundary membrane) (42). The connection between endothelial cells directly determines the permeability of BBB (43). For astrocytes, it not only forms a secondary barrier that further restricts entry of peripheral immune cells into CNS, but also maintains the integrity of BBB through self-secreting cytokines (43–46). When encountering disease, astrocytes can promote the differentiation of endothelial cells by secreting cytokines such as TGF-β, GNDF, bFGF, VEGF, angiopoietin, and then promote tissue repair furtherly (38, 47–51). In MS/EAE, sonic hedgehog (shh) was largely expressed in astrocytes (24, 52) and shh from astrocytes can act on endothelial cells by receptor ptch1, reducing their permeability and repairing BBB (53, 54). In our study, we were the first to demonstrate that HMGB1 could promote shh release in astrocytes.

Due to its sulfhydryl in C23, C45 and C106 are easily oxidized by active oxygen, HMGB1 has different isoforms. In EAE, the level of reactive oxygen increased on the peak stage and decreased on the chronic stage (55, 56), indicating it was highly possible that the redox state of HMGB1 varied in EAE. HMGB1 under different redox state would bind to different receptors and play different roles (13, 14). To further investigate the underling mechanism of shh release promoted by HMGB1, we used gene knockout mice. We noticed that spontaneous shh release from astrocytes existed and the level of shh in wild type astrocytes was higher than that in TLR2^-/-^, TLR4^-/-^, RAGE^-/-^ astrocytes. It indicated that the spontaneous release of shh was dependent on TLR2, TLR4 and RAGE. But the effect of HMGB1 on shh release was weakened only when RAGE receptor was knockout. Considering that gene knockout may bring unknown side effects, we further verified the above result using receptor blocking agents. Consistently, HMGB1 could not promote shh release after receptor RAGE was blocked. The result implied that the promoting effect of shh release from astrocytes *via* HMGB1 was mediated by RAGE. Together with other study that HMGB1 promoted axon growth by binding to RAGE in spinal cord injury (57), HMGB1 may play a protective role in disease *via* promoting shh release through receptor RAGE.

As for the possible signal pathway involved in HMGB1-RAGE axis, MAP kinases (JNK, p38, ERK) and stat3 were activated (58). In our results, only the addition of inhibitors for p38, JNK and stat3 could furtherly suppress the release of shh from astrocytes after HMGB1 administration. And the phosphorylation of p38, JNK and stat3 was inhibited in RAGE^-/-^ knockout astrocytes, compared to wild type astrocytes. The results indicated that the effect of HMGB1-RAGE-shh axis may be closely related with p38, JNK and stat3. Moreover, NF-κB signal pathway was also involved in HMGB1-RAGE axis in CNS and participated in neuroinflammation (59–61). It provided another possibility for HMGB1-RAGE-shh axis in astrocytes. Further researches are needed to explore the possibility.

In other reports, some agents attenuating MS/EAE was related to increased shh (62), we further proved it *via* shh protein treatment in EAE mice. Accordingly, shh treatment alleviated the progress of EAE, providing a direct evidence for its protective role in EAE. Considering the phenomenon of HMGB1 promoting shh release, HMGB1 may play a protective role in some way during the course of EAE. However, numerous study proved that HMGB1 exerted negative effects in EAE overall. Our data suggest a novel effect of HMGB1, providing a new understanding of DAMPs.

## Conclusion

HMGB1 promoted shh release from astrocytes through RAGE and its downstream (p38, JNK and stat3), which may indicated a new role of HMGB1 in EAE.

## Data Availability Statement

The raw data supporting the conclusions of this article will be made available by the authors, without undue reservation.

## Ethics Statement

The animal study was reviewed and approved by the Institutional Animal Care and Use Committee, Tongji Medical College, Huazhong University of Science and Technology.

## Author Contributions

FZ worked on conception and design. YFX performed the majority of the experiments. YS assisted to make EAE animal mode. WL, FFZ, JS, JL, HC, CT, YX, ZT, and FG contributed to the experimentation. YFX wrote the paper. FZ supervised the project, revised the manuscript, and financed the study. All authors contributed to the article and approved the submitted version.

## Funding

This work was supported by the grant awarded by the National Natural Science Foundation of China (Grant No. 31670876, 31470852) to FZ, and National Natural Science Foundation of China (Grant No. 82001281) to YFX.

## Conflict of Interest

The authors declare that the research was conducted in the absence of any commercial or financial relationships that could be construed as a potential conflict of interest.

## Supplementary Material

Supplementary Figure 1 The effect of HMGB1 (2μg/ml) stimulation on cell viability. Data are shown as mean ± SD.

Supplementary Figure 2 The change of total TLR4 protein expression in astrocytes after HMGB1 (2μg/ml) stimulation was analyzed by Western blot. Data are shown as mean ± SD (****P* < 0.001).

Supplementary Figure 3 The variation tendency of phosphorylation-**(A)** ERK, **(B)** stat3, **(C)** p38 and **(D)** JNK after HMGB1 (1μg/ml) stimulation for different time. Data are shown as mean ± SD (**P* < 0.05 compared with 0 min).

Supplementary Figure 4 The blocking effect of JNK blocker (SP 600125), stat3 blocker (SH-4-54), ERK blocker (SCH 772984) and p38 blocker (SB 203580) in different concentrations after HMGB1 (1μg/ml) stimulation for 10 min.
