# Supplementary figures and images for: HMGB1 Promotes the Release of Sonic Hedgehog From Astrocytes
Source: Front Immunol. 2021 Apr 1;12:584097. doi: 10.3389/fimmu.2021.584097 (PMC8047406; doi:10.3389/fimmu.2021.584097)

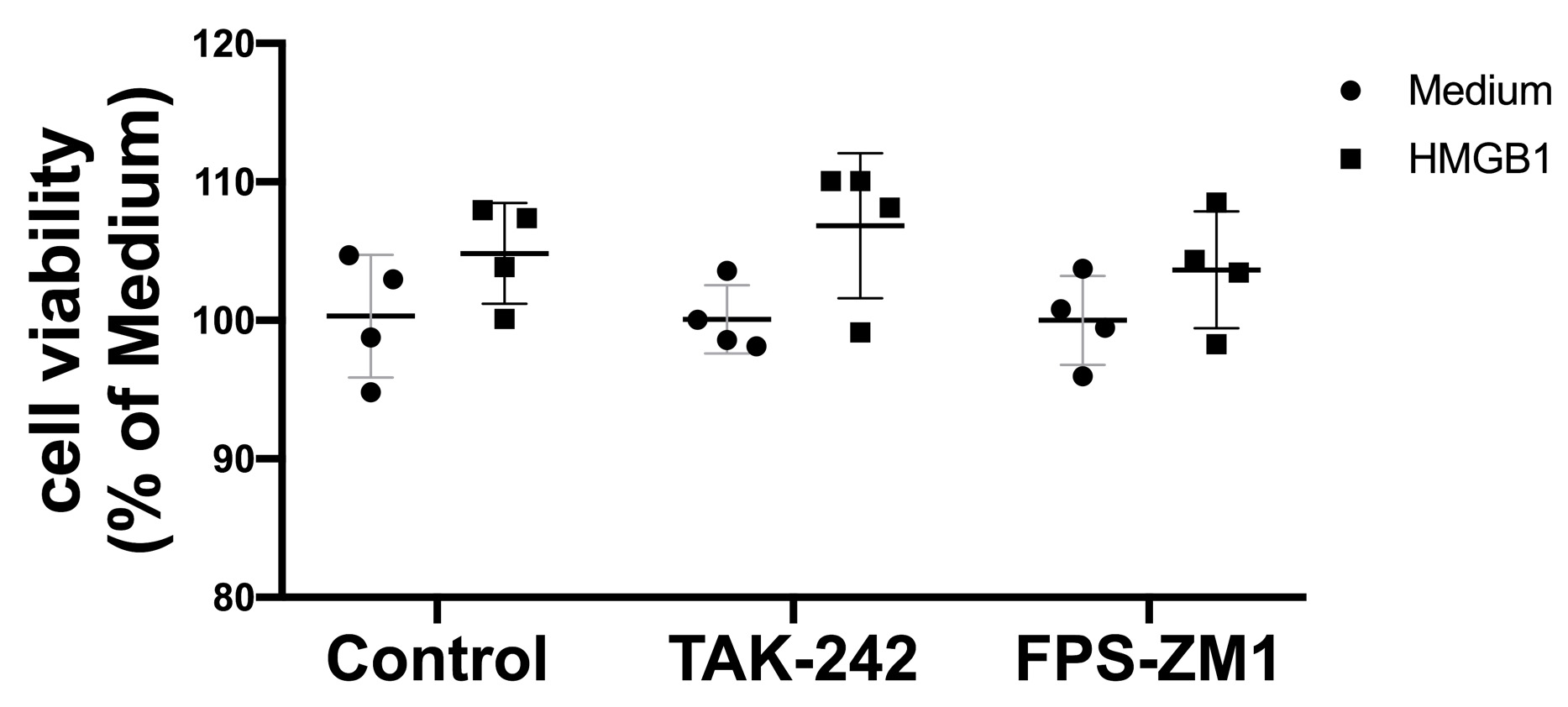

Supplement: Supplementary Figure 1 — The effect of HMGB1 (2μg/ml) stimulation on cell viability. Data are shown as mean ± SD. [file Image_1.jpeg]

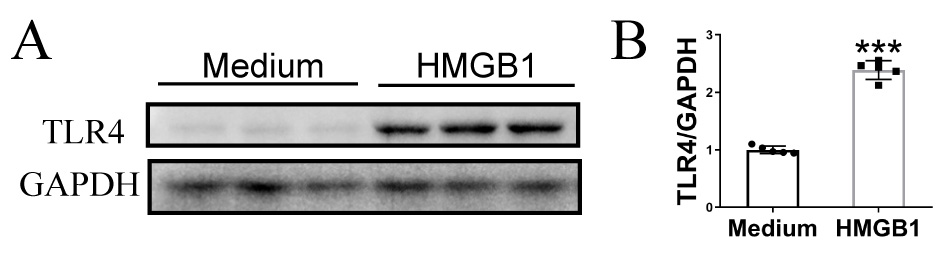

Supplement: Supplementary Figure 2 — The change of total TLR4 protein expression in astrocytes after HMGB1 (2μg/ml) stimulation was analyzed by Western blot. Data are shown as mean ± SD (***P < 0.001). [file Image_2.jpeg]

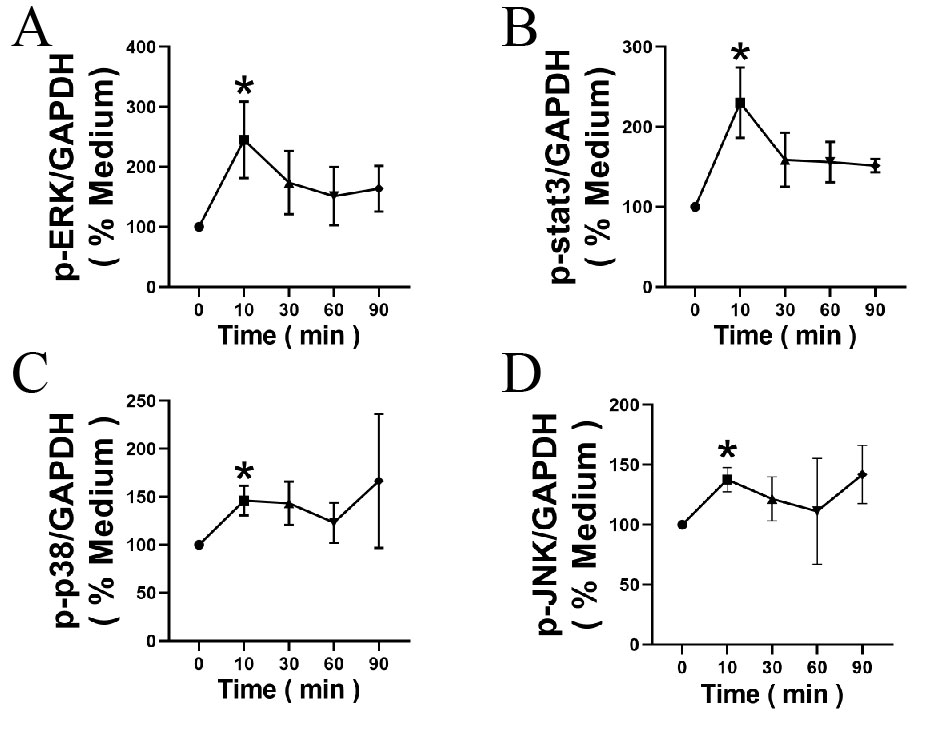

Supplement: Supplementary Figure 3 — The variation tendency of phosphorylation-(A) ERK, (B) stat3, (C) p38 and (D) JNK after HMGB1 (1μg/ml) stimulation for different time. Data are shown as mean ± SD (*P < 0.05 compared with 0 min). [file Image_3.jpeg]

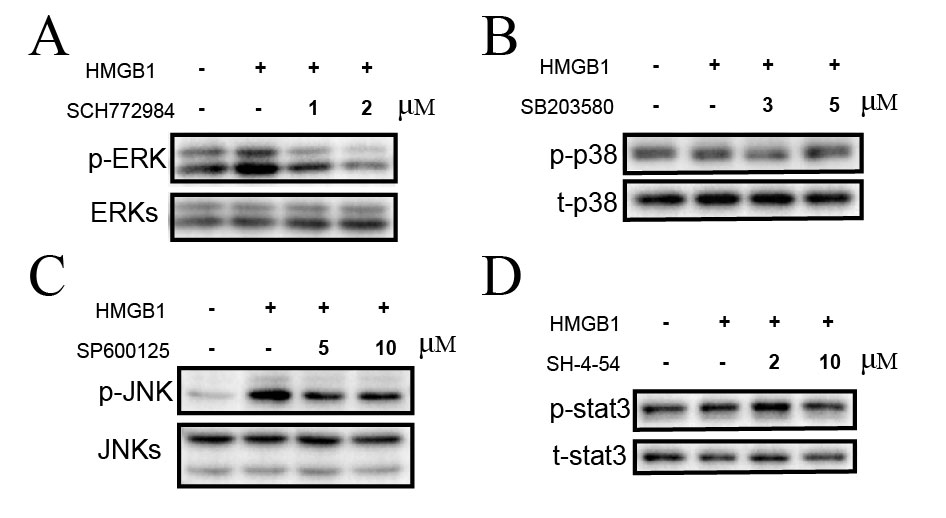

Supplement: Supplementary Figure 4 — The blocking effect of JNK blocker (SP 600125), stat3 blocker (SH-4-54), ERK blocker (SCH 772984) and p38 blocker (SB 203580) in different concentrations after HMGB1 (1μg/ml) stimulation for 10 min. [file Image_4.jpeg]
